# Microbiota, natural products, and human health: exploring interactions for therapeutic insights

**DOI:** 10.3389/fcimb.2024.1371312

**Published:** 2024-07-05

**Authors:** Hafsa Qadri, Abdul Haseeb Shah, Abdullah Almilaibary, Manzoor Ahmad Mir

**Affiliations:** ^1^ Department of Bioresources, School of Biological Sciences, University of Kashmir, Srinagar, India; ^2^ Department of Family and Community Medicine, Faculty of Medicine, Al Baha University, Al Bahah, Saudi Arabia

**Keywords:** microbiota, gut microbiome, anaerobic bacteria, microbial transformation, natural products, human health

## Abstract

The symbiotic relationship between the human digestive system and its intricate microbiota is a captivating field of study that continues to unfold. Comprising predominantly anaerobic bacteria, this complex microbial ecosystem, teeming with trillions of organisms, plays a crucial role in various physiological processes. Beyond its primary function in breaking down indigestible dietary components, this microbial community significantly influences immune system modulation, central nervous system function, and disease prevention. Despite the strides made in microbiome research, the precise mechanisms underlying how bacterial effector functions impact mammalian and microbiome physiology remain elusive. Unlike the traditional DNA-RNA-protein paradigm, bacteria often communicate through small molecules, underscoring the imperative to identify compounds produced by human-associated bacteria. The gut microbiome emerges as a linchpin in the transformation of natural products, generating metabolites with distinct physiological functions. Unraveling these microbial transformations holds the key to understanding the pharmacological activities and metabolic mechanisms of natural products. Notably, the potential to leverage gut microorganisms for large-scale synthesis of bioactive compounds remains an underexplored frontier with promising implications. This review serves as a synthesis of current knowledge, shedding light on the dynamic interplay between natural products, bacteria, and human health. In doing so, it contributes to our evolving comprehension of microbiome dynamics, opening avenues for innovative applications in medicine and therapeutics. As we delve deeper into this intricate web of interactions, the prospect of harnessing the power of the gut microbiome for transformative medical interventions becomes increasingly tantalizing.

## Graphical Abstract

Depiction of the intricate interplay between gut microbiota and human health, emphasizing the influence of food and drugs on the gut microbiome, microbial transformation of gut compounds, and the exploration of consequential human microbial metabolites (Created with Biorender).

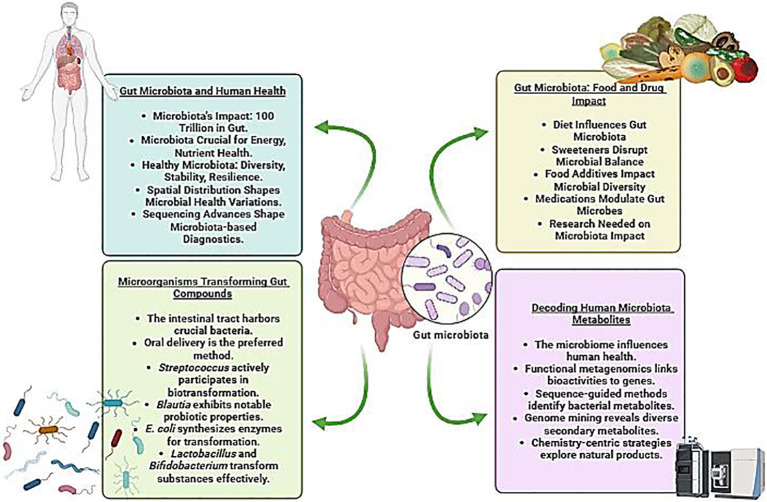

## 1 Introduction

**Figure 1:**
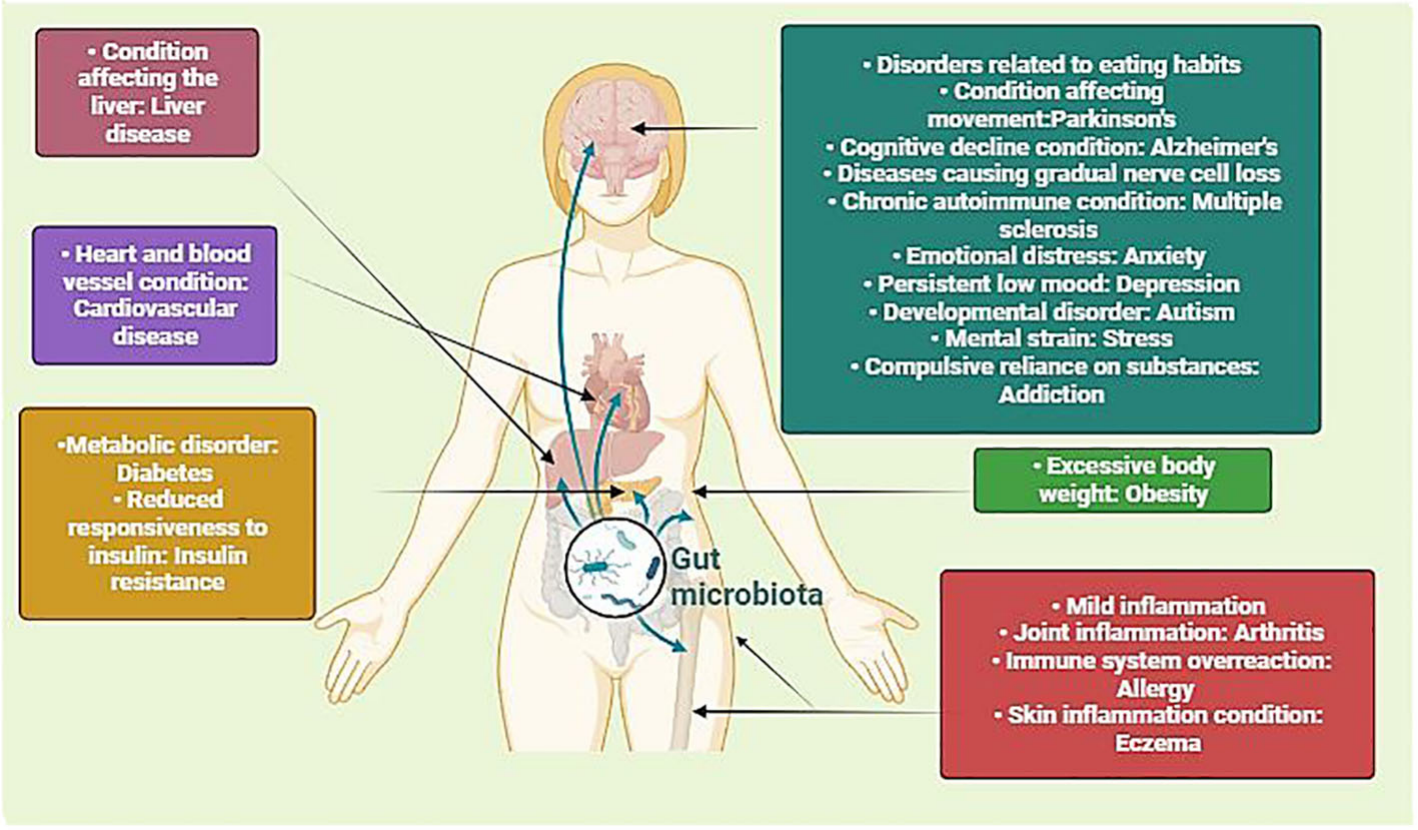
Illustration depicting the intricate interplay between microbiota and human physiology, impacting different health conditions. By portraying how alterations in the gut microbiota can impact diverse physiological processes, including metabolism, immune function, and neurobiology, it underscores the significance of this interplay in both health and disease. Understanding these interactions is crucial for developing targeted interventions to modulate the microbiota and promote optimal health outcomes across diverse populations. (Created with Biorender).

The human microbiome encompasses the collective genetic material of microorganisms residing within us, including protozoa, archaea, eukaryotes, viruses, and primarily bacteria, which coexist symbiotically on and inside various regions of the human body. These inhabited sites include the mouth, reproductive organs, airways, skin, and digestive system (Lloyd-Price et al., 2016). Interestingly, the human digestive system harbors a thriving ecosystem of microbes collectively referred to as the microbiota, comprising an astonishing ∼10^13^-10^14^entities. This intricate assembly, primarily composed of anaerobic bacteria, encompasses 500–1,000 distinct species with a genetic repertoire estimated to surpass the human genome by a factor of 150 (Kho and Lal, 2018; Marsh et al., 2023; Walker and Hoyles, 2023). Functioning as a metabolic entity finely attuned to human physiology, the microbiota operates as an organ, orchestrating processes that necessitate no evolutionary adaptations on our part. Among its pivotal functions is the processing of dietary components that would otherwise be indigestible like plant polysaccharides (Bäckhed et al., 2004). The human microbiome has emerged as a linchpin in complex physiological processes, influencing a spectrum ranging from immune system modulation to the central nervous system and brain development (Kau et al., 2011; Smith, 2015). Insights derived from mouse models underscore the indispensable part of a healthy bacterial environment in maintaining typical physiological processes, in contrast to imbalances, or dysbiosis, linked to an array of diseases including cancer, diabetes, obesity, and colitis (**Figure 1**) (Garrett et al., 2007; Ridaura et al., 2013; Smith et al., 2013; Bongers et al., 2014). The burgeoning field of microbiome research is further underscored by a spike in clinical trials and venture capital investments, with over 1,200 clinical trials related to the “gut microbiome” registered in the National Institutes of Health clinical trials database (Gormley, 2016; Marchesi et al., 2016).

There is still a significant knowledge vacuum regarding the precise mechanisms by which bacterial functions, also known as effector functions, influence mammalian or microbiome physiology, despite growing evidence linking host-associated bacteria to both healthy development and disease in animal models as well as correlational findings in humans. The traditional framework of molecular biology, which follows the linear flow of genetic information from DNA to RNA to protein, may not fully capture the complexity of biological systems. In many cases, biological functions do not solely result in protein synthesis but also involve the generation and utilization of small molecules. This phenomenon is particularly evident in bacteria, where interactions with the environment heavily rely on low-molecular-weight compounds, including small molecules and natural products. While these small molecules are not directly encoded by DNA, they play crucial roles in mediating microbial functions and their interactions with the host organism. Therefore, to comprehensively understand the molecular mechanisms underlying the role of the human microbiome in both health and disease, it is essential to thoroughly identify and characterize the small compounds produced by human-associated bacteria (Milshteyn et al., 2018).

Gut microbes perform a pivotal function in decomposing and transforming natural products, generating a diverse array of metabolites and functional compounds having unique physiological functions that the host organism cannot synthesize on its own (Koppel et al., 2017; Xie et al., 2020). The process of microbial transformation in natural products encompasses various chemical reactions, including demethylation, hydrolysis, methylation, etc, which collectively regulate the form of the natural product substrates (Morgan et al., 2022; Rocchetti et al., 2022). Importantly, the impact of gut microbiota extends beyond mere structural modifications, significantly influencing the chemical landscape, pharmacological activities, and metabolic mechanisms of natural products. Surprisingly, the ability to harness gut microorganisms for the massive manufacture of active metabolites and compound synthesis remains largely unexplored. Delving into the study of these gut microorganisms, their metabolites, and the intricate reactions engaged in the dynamic interplay between gut microbiota and natural products holds immense importance. Unraveling these interactions is essential to both realizing the promise of natural products in several applications and comprehending the pharmacological processes at work (Zhao et al., 2022). This exploration opens avenues for further research into the utilization of gut microbes in the synthesis of bioactive compounds on a larger scale, shedding light on innovative approaches for the production and utilization of these compounds. Consequently, studying the multifaceted relationships between natural products and gut microbiota not only enhances our understanding of pharmacological mechanisms but also paves the way for harnessing the therapeutic potential of these interactions.

Our goal in this review study is to explore and synthesize the present state of information in this dynamic field, shedding light on the interactions between natural products, bacteria, and human health. Recent efforts have illuminated a fascinating facet of microbiome dynamics: the generation of natural products. This burgeoning area of research explores metabolites derived from the microbiome and seeks to unravel their part in human health (Wilson and Nicholson, 2017). In this review, we delve into these recent efforts, describing key approaches used to identify and characterize microbiome-derived natural products. This comprehensive exploration contributes to the evolving understanding of the intricate relationships between the microbiome, natural products, and human health, ultimately paving the way for innovative applications in medicine and therapeutics.

## 2 Microbiome

Microbial organisms commonly exist in communal arrangements and often form close associations with intricate beings like humans, forming mutualistic, parasitic, pathogenic, and other associations. The ensemble of these microorganisms is referred to as the microbiome or microbiota. While the term “microflora” has been used, it is considered a misnomer as “flora” traditionally represents the plant kingdom (Liang et al., 2018). Initially, the term “microbiome” denoted the group of microbes and the contents of their genomes, while “microbiota” described the microbial community within their hosts. However, the interchangeability of “microbiome” and “microbiota” has become prevalent (Ursell et al., 2012). The human body has a microbiome in every part of it, from the skin to the gut and even in places like the bloodstream that were thought to be sterile in the past (Proal et al., 2014). Numerous reports suggest the presence of over 10,000 microbial species occupying various human body parts (Blaser, 2006; Ley et al., 2006). Although skin and vaginal sites exhibit comparatively minimal diversity of microbes, greater diversity is observed in sites such as the gut (Jiang et al., 2009). There can be a significant relationship between human diseases and the microbiome and vice versa. For instance, long-term lung conditions may change the lung microbiome’s makeup, which may then impact host immunity and defense and worsen the conditions (O’Dwyer et al., 2016). Furthermore, research has revealed that the microbiome present in the blood or lungs is affected by the presence of infection (Jiang et al., 2009; Jostins et al., 2012; Kwan et al., 2013; Leung and Wu, 2015).

### 2.1 Gut microbiome

The gut microbiome, comprising the genetic material of microorganisms like fungi, protozoa, bacteria, etc, resides in the digestive tracts of humans and other animals (**Table 1**), including insects (Liang et al., 2018). Having evolved alongside its host for millennia, the human gut microbiome plays a crucial role in various essential activities, including nutrient absorption and digestion (Turnbaugh and Gordon, 2009; Hehemann et al., 2010), detox and body defense (Relman, 2012), host immune system development (Turnbaugh and Gordon, 2009), etc. The mammalian gut hosts a diverse array of microbes, with the majority belonging to the Firmicutes and Bacteroidetes (Ley et al., 2008). This pattern holds correct across different populations, such as Koreans (Nam et al., 2011), Africans (De Filippo et al., 2010; Schnorr et al., 2014), Europeans and Americans (Qin et al., 2010), and Danes though not in the case of Chinese (Wu G. et al., 2014). The diversity of microorganisms can carry distinct consequences for diseases in various populations. For instance, individuals with type 2 diabetes from European and Chinese backgrounds exhibit varying compositions of gut microbiomes, with the Chinese population showing greater species diversity (Qin et al., 2012). Nonetheless, understanding the significant differences among these populations, considering factors like age, environment, and genetics, requires further investigation (Tai et al., 2015).

**Table 1:** Primary categories of gut microbiota in both humans and animal models (Hillman et al., 2017).

S. No	Category	Human	Mouse	Rat
**1.**	**Bacteria**	*Proteobacteria*		
		*Actinobacteria*		
		*Bacteroidetes*	*Bacteroidetes*	*Bacteroidetes*
		*Firmicutes*	*Firmicutes*	*Firmicutes*
**2.**	**Eukarya**	*Cladosporium*	*Zygomycota*	*Zygomycota*
		*Saccharomyces*	*Chytridiomycota*	*Chytridiomycota*
		*Candida*	*Ascomycota*	*Ascomycota*
		*Malassezia*	*Basidiomycota*	*Basidiomycota*
**3.**	**Archaea**	*Nitrososphaera*		
		*Methanobrevibacter*	*Methanobrevibacter*	*Methanobrevibacter*
**4.**	**Viruses**	*Adenoviridae*		
		*Polyomaviridae*		
		*Papillomaviridae*		
		*Herpesviridae*	Variable	Variable

The gut microbiome harbors millions of diverse microorganisms, each contributing to its metabolic diversity and adaptability, with some genes potentially acquired from environmental bacteria (Hehemann et al., 2010; Qin et al., 2010). Notably, three primary enterotypes [Enterotypes are defined as clusters or patterns of microbial community composition in the gut microbiome that are driven by various factors such as diet, geography, host genetics, and lifestyle. Rather than discrete and stable classifications, enterotypes are now viewed as dynamic and context-dependent configurations of the gut microbiota. These configurations may shift over time in response to environmental changes, host health status, and other factors, reflecting the complexity and flexibility of the gut microbial ecosystem (Costea et al., 2018)]—Bacteroides, Prevotella, and Ruminococcus—have been identified in the human gut across diverse populations (Arumugam et al., 2011). These enterotypes, observed in Europeans, Japanese, and Americans, demonstrate a consistent presence in mice and chimpanzees (Moeller et al., 2012; Hildebrand et al., 2013; Wang et al., 2014). While the composition is mainly shaped by the host’s evolution, recent findings suggest that diet has a more significant impact on the metabolome than the microbiome. Discrepancies, especially regarding the prevalence of ecotypes like Ruminococcus, warrant further investigation considering factors such as sample size and variations in sampling methods (Arumugam et al., 2011; Wu et al., 2011; Huse et al., 2012; Zupancic et al., 2012; Gorvitovskaia et al., 2016; Liang et al., 2017). The gut microbiota comprises autochthonous microbes (Autochthonous refers to indigenous or native microbial species that are typically well-adapted to a specific environment, such as the gut) residing on the colonic mucosa epithelium and allochthonous microbes (Allochthonous refers to microbial species that are not native to a particular environment but are introduced from external sources) transiently passing through the lumen with digesta (Schnabl and Brenner, 2014). Distinguishing between these “residents” and “passengers” becomes crucial, as their roles are believed to differ significantly. The ratio of autochthonous to non-autochthonous microbes serves as a valuable indicator for assessing the progression of cirrhosis (Bajaj et al., 2014).

Diet and phylogeny are major contributors to the modification of the gut microbial community in various species, including mammals (Ley et al., 2008; Miyake et al., 2015). Genome-scale metabolic modeling reveals that changes in the host diet significantly alter the makeup of key human gut bacteria (eg. *B. thetaiotaomicron*) (Shoaie et al., 2013). Examples such as the impact of alcohol on intestinal microbiota emphasize the bidirectional relationship between diet and microbial composition, influencing host metabolism and related diseases (Mutlu et al., 2009; Chen et al., 2011; Yan et al., 2011; Mutlu et al., 2012; Queipo-Ortuño et al., 2012). Host genetics also play a crucial role in determining microbiome composition (Garrett et al., 2007; Maslowski et al., 2009; Benson et al., 2010; Brinkman et al., 2011; Lepage et al., 2011; Hashimoto et al., 2012; McKnite et al., 2012; Goodrich et al., 2014; Knights et al., 2014; Benson, 2015; Kubinak et al., 2015; Bonder et al., 2016a; Snijders et al., 2016; Igartua et al., 2017). The identification of shared susceptibility loci between inflammatory bowel disease and specific infectious organisms underscores the significance of exploring the interconnections among susceptibility, microbiome composition, and the development of the disease. This emphasizes the need to develop effective protocols for disease prevention by understanding these relationships (Kane et al., 2011; Henao-Mejia et al., 2012; Jostins et al., 2012; Knights et al., 2014). In the gut, Gram-negative bacteria produce lipopolysaccharide (LPS), a microbial product transported with chylomicrons (Balagopal et al., 2008; Jiang et al., 2009; Hofer et al., 2010; Marchetti et al., 2013). LPS acts as a potent stimulator of innate immunity, with its levels serving as important indicators for survival in peritoneal dialysis patients (Kwan et al., 2013). Similarly, trimethylamine (TMA) and its oxidation product, trimethylamine N-oxide (TMAO), impact patient morbidity, highlighting the far-reaching consequences of localized microbiome activity (Tang et al., 2017). These observations underscore the potential of using plasma levels of LPS and bacterial DNA as markers for both systemic inflammation and prognosis (Alexander and Rietschel, 2001).

## 3 Gut microbiota and human health

**Figure 2:**
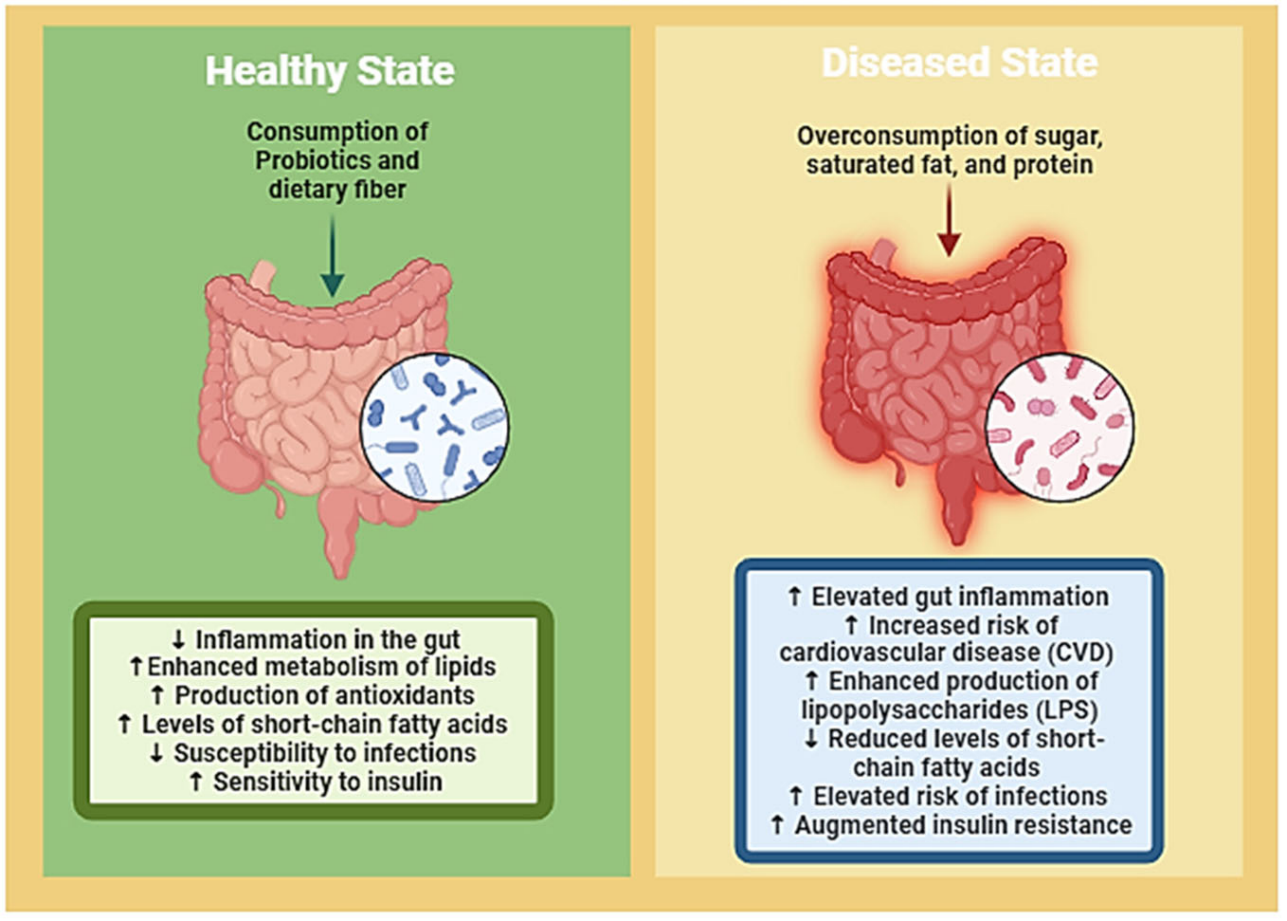
Visual representation delineates the intricate interplay between gut microbiota, nutrition, and the delicate balance between health and disease states. It illustrates how dietary factors can either promote a healthy gut microbiota composition, which contributes to overall well-being, or lead to dysbiosis, predisposing individuals to various diseases. Through highlighting this dynamic relationship, the figure underscores the potential of targeted dietary interventions to restore microbial balance and mitigate disease risk, thereby emphasizing the crucial role of nutrition in maintaining gut health and preventing the onset of illness. (Created with Biorender).

The GI tract of humans houses a vast community of microbes of approximately 100 trillion microorganisms, significantly impacting human health and disease (**Figure 2**). This intricate association between gut microbiota and fundamental biological mechanisms has been extensively studied, revealing its involvement in immune systems, metabolic processes, and nutrient absorption (Ley et al., 2006). The gut microbiota, comprising bacteria, yeasts, and viruses, plays a crucial role in energy and nutrient extraction through diverse metabolic genes, providing unique enzymes and biochemical pathways. Additionally, it contributes to the manufacture of essential substances like lipids, vitamins, and amino acids (Rowland et al., 2018). In terms of immunity, the human microbiota acts as a defense mechanism by producing antimicrobial substances and influencing intestinal mucosal growth as well as the immune system’s development. When the gut microbiota is in a healthy state, it interacts symbiotically with the host and is stable and resilient. Defining a “healthy” gut microbiota involves considering factors such as high taxonomic diversity, microbial gene richness, and a stable core microbiota (Fan and Pedersen, 2021). Nevertheless, individual variations and dynamic changes influenced by aging, and contextual elements, including the use of medications, are noted.

Spatial distribution within the GI tract also contributes to microbial variation, with distinct microbial communities present in the small intestine and colon due to differences in transit time, bile concentration, flow rates, and pH (Flint et al., 2012). Age-related variations indicate an increase in microbiota diversity from childhood to adulthood, followed by a decrease in older age, where shifts in microbial composition, including decreased Bifidobacterium and increased Clostridium and Proteobacteria, are observed (Rinninella et al., 2019). The impact of microbiota on human well-being extends beyond compositional studies. Recent advancements in high-throughput sequencing, microbiota interaction modeling, and simulation techniques have shifted focus toward understanding the causality of microbiota functions. This has significant implications for the advancement of microbiome-based diagnostics and customized medicine (Ley et al., 2006; Bouskra et al., 2008; Amabebe et al., 2020).

Therefore, the intricate interplay between gut microbiota and human health involves dynamic mechanisms influenced by age, spatial distribution within the GI tract, and environmental factors. As research progresses, a deeper understanding of microbiota’s functions is essential to strengthen the advancement of personalized medicine and microbiome-based diagnostics.

### 3.1 The impact of food and drugs on the composition of the gut microbiota

The quantity of gut bacteria is greatly influenced by particular foods and dietary habits, which in turn affects health. High-intensity sweeteners, widely utilized as sugar substitutes, raise concerns based on animal studies. Despite regulatory agencies deeming them “generally recognized as safe,” sucralose, aspartame, and saccharin disrupt gut microbiota balance and diversity. Rats exposed to sucralose for twelve weeks displayed elevated amounts of total aerobic bacteria, Bacteroides, and Clostridium, along with a significantly higher fecal pH (Abou-Donia et al., 2008). Mice exposed to sucralose for six months exhibited increased expression of pro-inflammatory genes and altered fecal metabolites (Bian et al., 2017). Common food additives that impact gut flora include emulsifiers, which are included in processed meals. Mice given carboxymethylcellulose and polysorbate-80 displayed decreased microbial diversity, decreased Bacteroidales and Verrucomicrobia, and enrichment of inflammation-promoting Proteobacteria (Chassaing et al., 2015). Concerns extend to certain restrictive diets. Studies on vegan diets revealed variations in the gut microbe-produced serum metabolites, but only slight alterations in the bacterial communities (Wu G. et al., 2014). Gluten-free diets, recommended for gluten sensitivity or celiac disease, altered gut microbiota profiles in healthy individuals, potentially affecting useful microbial species (Bonder et al., 2016b). The low FODMAP diet, beneficial for irritable bowel syndrome, led to significant microbiota and metabolome changes (McIntosh et al., 2016; Gibson, 2017; Halmos, 2017; Bennet et al., 2018). Medications also modulate the composition of gut flora. An important research identified drugs such as progesterone, rupatadine, TNF-α inhibitors, and osmotic laxatives, as major modulators (Falony et al., 2016). Proton pump inhibitors and antibiotics, extensively used in humans and livestock, influence gut microbes, potentially contributing to obesity (Jackson et al., 2015; Blaser, 2016).

**Figure 3:**
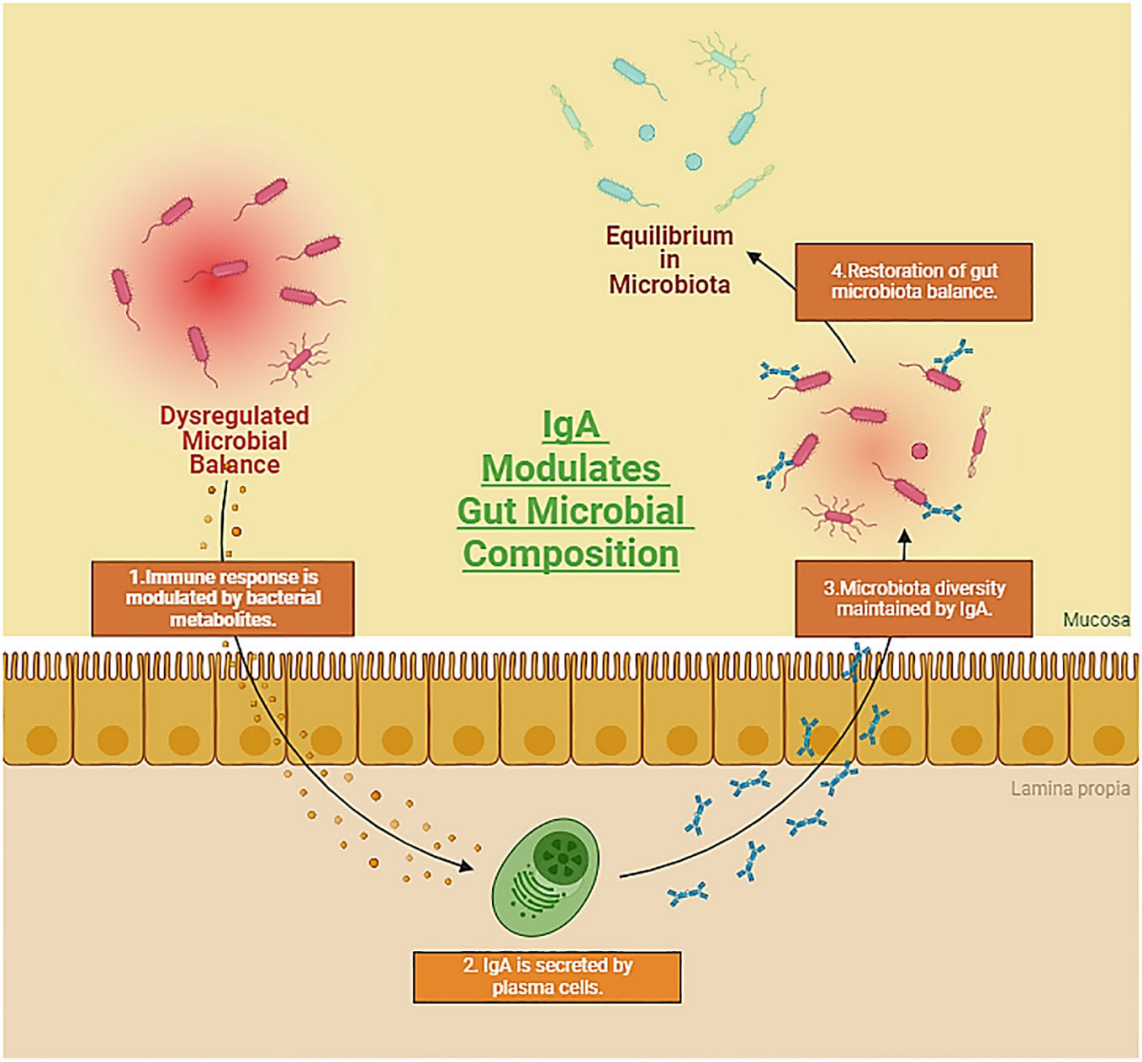
Representation showcasing the symbiotic relationship between the gut microbiota and autoimmune diseases, while emphasizing the crucial role of Immunoglobulin A (IgA) in preserving colonic homeostasis. The dynamic interplay between host immune responses and the gut microbiome, coupled with IgA-mediated regulation, underscores the intricate mechanisms influencing autoimmune processes and maintaining gastrointestinal equilibrium. (Created with Biorender).

While clinical evidence is insufficient for clear recommendations, future studies on food additives, drugs, and dietary modifications should consider their effects on the gut microbiota. In medical contexts like cancer treatment and autoimmune disorders (**Figure 3**), where microbiota changes impact responses, understanding these dynamics becomes crucial (Alexander et al., 2017). Animal studies also highlight the role of specific gut microbes in transforming compounds for protective effects, emphasizing the interconnectedness of diet, gut health, and overall well-being (Spanogiannopoulos et al., 2016).

## 4 Crucial gut microorganisms involved in the transformation of natural compounds

Oral administration is the preferred mode of drug delivery, as evidenced by the 84% oral composition of the top 50 best-selling pharmaceuticals in the US and Europe (Vinarov et al., 2021). The impact of gut microbiota on the durability of orally administered natural products has garnered significant interest in recent years. The intestinal tract harbors a plethora of bacteria crucial for normal digestive function, with approximately 98% of gut microorganisms in healthy individuals falling into 4 phyla: *Proteobacteria*, *Actinobacteria*, *Firmicutes*, and *Bacteroidetes* (Manor et al., 2020; Ye et al., 2021; de Vos et al., 2022). Specific gut microorganisms, like *Escherichia coli*, *Streptococcus*, etc. actively take part in the biotransformation of natural products. The resulting metabolites contribute to enhanced intestinal absorption, playing a significant pharmacological role (Zhao et al., 2022).

### 4.1 Streptococcus

The *Streptococcus* species, characterized by gram-positive, circular, or ovoid cells organized in chains or pairs, is commonly found in the nasopharynx and human stool (Lannes-Costa et al., 2021). Meta-transcriptomic study reveals that *Streptococcus* predominantly expresses the phosphotransferase system, suggesting their primary role in utilizing the carbohydrates that are present in the small intestine (Zoetendal et al., 2012). *Streptococcus LJ-22* expresses β-glucuronidase activity, converting GL to 18β-glycyrrhetinic acid-3-O-β-D-glucuronic acid (GAMG), known for its anti-allergic properties. This compound has demonstrated anti-allergic potential against LPS-induced *RAW264.7* cells (Guo et al., 2018). Furthermore, tannic acid degradation by *Streptococcus gallolyticus subsp. Gallolyticus* may contribute to colorectal cancer development by neutralizing its toxicity in the case of tumor cells (Oehmcke-Hecht et al., 2020). *Streptococcus thermophilus GIM 1.321* demonstrates high β-glucosidase production, facilitating the breakdown of fructus anthocyanins into beneficial compounds like ferulic acid, CAA (caffeic acid), and CHA (chlorogenic acid) (Cheng et al., 2016). Administering CAA and CHA from these processes has shown the potential to lower blood pressure and provide antioxidant effects (Agunloye et al., 2019). *Streptococcus* strains, acting as commensals, pathogens, or opportunistic pathogens in the gut, require further exploration regarding their impact on the well-being of humans. Comprehending the way *Streptococcus* metabolizes natural compounds may help regulate the gut microbiota and improve the effectiveness of treatments. In-depth *in vivo* research is crucial to find out if focusing on bacteria that break down tannic acid could help develop more potent treatments for colorectal cancer. Overall, unraveling the intricacies of *Streptococcus* metabolism holds promise for advancing our comprehension of the gut microbiota and optimizing treatment interventions (Zhao et al., 2022).

### 4.2 Blautia

Blautia species, prevalent in the anaerobic environments of mammalian guts and feces, exhibit notable probiotic properties in the biotransformation of natural products. Tremaroli and Bäckhed (2012) (Tremaroli and Bäckhed, 2012) highlight *Blautia’s* involvement in flavonoid processing, encompassing demethylation, O- and C-deglycosylation, and C-ring cleavage catalyzed by enzymes like O-glycosidase and β-glucosidases (Braune et al., 2016). Notably, *Blautia* sp. *MRG-PMF1* demonstrates hydrolytic prowess, conver ting compounds like 5,7-dimethoxyflavone into chrysin and 5,7,4-trimethoxyflavone into apigenin. This strain also exhibits deglycosylation activity on various isoflavones, flavones, and flavonols, transforming them into their corresponding aglycones (Kim et al., 2014). In anaerobic environments, *Blautia* sp. *MRG-PMF1* catalyzes curcumin to generate demethoxycurcumin, which has anti-inflammatory and anti-cancer characteristics and further metabolizes icariin into desmethylicaritin, which has estrogenic properties (Wu H. et al., 2016; Burapan et al., 2017; Hatamipour et al., 2019). Another strain, *Blautia* sp. *AUH-JLD56*, exclusively biotransforms arctiin or arctigenin into demethylated products with enhanced antioxidant potential (Liu et al., 2013). The creation of novel enzymes and bioactive metabolites is greatly encouraged by the increasing scholarly interest in *Blautia’s* involvement in the biotransformation and metabolism of herbal plants and functional foods (Meng et al., 2020).

### 4.3 E. coli

*Escherichia coli*, a facultative anaerobic, non-sporous, gram-negative bacterium, predominantly resides in vertebrate intestines, playing a crucial role in glycosidase production for the transformation of exogenous substances (Foster-Nyarko and Pallen, 2022). Notably, *E. coli* strain *HGU-3* synthesizes β-glucuronidase, facilitating the conversion of baicalin to baicalein, which exhibits superior anti-oxidant and anti-inflammatory potential (Han et al., 2016; Li D. et al., 2019). Additionally, certain *E. coli* strains, like *DH10B*, demonstrate high curcumin-converting activity through the expression of NADPH-dependent curcumin/dihydrocurcumin reductase (CurA) (Hassaninasab et al., 2011; Tan et al., 2014). The resulting dihydrocurcumin (DHC) and tetrahydrocurcumin (THC) show promising therapeutic benefits in hepatic steatosis, surpassing the effects of curcumin (Chen et al., 2018; Yu et al., 2018).

Moreover, E. coli strains *Nu*, *MC*, and *WC-1* exhibit cinnamyl esterase properties, releasing hydroxycinnamic acids with *in-vitro* and *in-vivo* anticancer and antioxidant potential (Zhao et al., 2022). Understanding the genetic and biochemical aspects of *E. coli* opens avenues for synthesizing natural product derivatives with diverse health benefits. This knowledge is pivotal for exploring the potential of *E. coli* in producing compounds such as DHC (dihydrocaffeic acid) and THC (tetrahydrocurcumin), which effectively regulate triglyceride levels and demonstrate novel therapeutic advantages in hepatic steatosis. Overall, comprehending the capabilities of *E. coli* contributes to the development of natural product derivatives with diverse health benefits (Chen et al., 2018; Yu et al., 2018; Rodríguez-Daza et al., 2021; Candeliere et al., 2022).

### 4.4 Eubacterium

The genus *Eubacterium*, a gram-positive constituent of the human gut microbiota, plays a vital part in metabolizing various substances (Mukherjee et al., 2020). Notably, *E. ramulus*, a well-studied flavonoid-degrading bacterium in the human intestine, produces enzymes like chalcone isomerase and flavanone-/flavanonol-cleaving reductase (Gall et al., 2014). These enzymes break down flavonoids into dihydrochalcone and its derivatives, known for their anti-inflammatory and antioxidant effects. *E. ramulus* strain wK1 further degrades flavonol quercetin and flavone luteolin, converting them into specific acids through reduction and ring fission (Zhao et al., 2022).

Another strain, *E. cellulosolvens ATCC 43171T*, is implicated in deglycosylating flavonoid O- and C-glucosides, exclusively catalyzing the deglycosylation of C-glucosides (Braune and Blaut, 2012; Braune et al., 2016). Additionally, *Eubacterium L-8* exhibits the ability to hydrolyze terpenoid glycyrrhizin into 18β-glycyrrhetinic acid (18β-GA), which, in turn, demonstrates anti-inflammatory effects in preventing airway allergic inflammation (Liu et al., 2022). While these metabolic transformations highlight the potential health benefits of *Eubacterium* spp., it is emphasized that further *in vivo* studies are imperative to fully comprehend and harness the diverse advantages offered by this genus. This research could unlock opportunities to maximize the positive impact of *Eubacterium* strains on human health (Zhao et al., 2022).

### 4.5 Lactobacillus

The genus *Lactobacillus*, classified within the phylum *Firmicutes*, plays a crucial role in maintaining microbial balance and safeguarding gastrointestinal mucosal integrity (Dempsey and Corr, 2022). Certain *Lactobacillus* species are equipped with an array of metabolic enzymes, including α-rhamnosidases, tannase, and gallate decarboxylases, enabling them to transform exogenous substances (Reverón et al., 2017; Li B-C. et al., 2019; Ferreira-Lazarte et al., 2021).

*L. rhamnosus* NCTC 10302, possessing both β-glucosidase and α-rhamnosidase activities, demonstrates the ability to convert hesperetin-7-O-rutinoside and naringenin-7-O-rutinoside into their respective aglycones and 3-(phenyl) propionic acid through hydrolysis, ring fission, and dehydroxylation (Pereira-Caro et al., 2018). In a similar vein, *L. plantarum* expresses tannase, facilitating the hydrolysis of gallate and protocatechuate esters, ultimately producing gallic acid (Jiménez et al., 2014). Gallic acid, present in concentrations of 11.5–46 μg/ml, exhibits a protective part against LPS-induced inflammation and oxidative stress by suppressing the MAPK/NF-κB pathway and activating the Akt/AMPK/Nrf2 pathway (Tanaka et al., 2018).

Fang et al. witnessed the production of gallic acid and pyrogallol through the breakdown of gallotannins by gallotannin-metabolizing enzymes in *L. plantarum* WCFS1, suggesting potential prebiotic-probiotic assocaitions in preventing diet-induced metabolic diseases (Reverón et al., 2015; Fang et al., 2019). Additionally, *Lactobacillus* sp. *Niu-O16* reduces daidzein to dihydrodaidzein with daidzein reductase activity, and dihydrodaidzein, at concentrations of 2.5–5 μM, supresses NF-κB activation and MAPK phosphorylation, therefore enhancing osteoporosis (Wang et al., 2007; Heng et al., 2019; Kim et al., 2019).

*L. casei*, *L. plantarum*, and *L. acidophilus* significantly impact the deglycosylation of piceid to resveratrol, enhancing bioavailability and bioactivity (Basholli-Salihu et al., 2016). Furthermore, feruloyl esterases from *L. reuteri*, *L. helveticus*, and *L. fermentum* hydrolyze chlorogenic acid, releasing caffeic acid (Santos et al., 2018). These results underscore the potential of *Lactobacillus* in health-improving pharmaceuticals and food products, but more investigation is required to clarify the fundamental transformation mechanisms (Zhao et al., 2022).

### 4.6 Bifidobacterium

*Bifidobacterium*, a widely distributed genus within the Actinobacteria phylum, serves as one of the initial colonizers in the human gut microbiota (Satti et al., 2021; He et al., 2023). Key species include *B. angulatum*, *B. breve*, *B. Catenulatum*, *B. adolescentis*, *B. longum* etc, collectively constituting less than 10% of the adult human microbiome yet playing a crucial role in host health (Turroni et al., 2008; Turroni et al., 2009; Hidalgo-Cantabrana et al., 2018). Some *Bifidobacterium* species express feruloyl esterase, enabling the generation of phenolic acids. For example, B. animalis’s feruloyl esterase can hydrolyze chlorogenic acid into caffeic acid (CAA) (Raimondi et al., 2015). CAA (10–30 mg/kg) has demonstrated hepatoprotective effects in mice, preventing acetaminophen-induced acute liver injury by enhancing Nrf2 transcription (Raimondi et al., 2015; Pang et al., 2016). *Bifidobacterium’s* involvement in the gut facilitates the metabolism of various compounds, including glycosides, saponins, and flavanones (Zhao et al., 2022).

Specific strains of *Bifidobacterium*, such as *B. longum R0175* and *B. longum SBT2928*, exhibit distinct metabolic activities. *B. longum R0175* facilitates the production of 3-(3′-hydroxyphenyl) propionic acid and 3-(phenyl) propionic acid from hesperidin by ring-cleavage and demethylation (Pereira-Caro et al., 2018). *B. longum SBT2928* contributes to bile acid metabolism by hydrolyzing main human and animal bile salts, suggesting a potential role in reducing cholesterol levels *in vivo* (Tanaka et al., 2000). Furthermore, *B. breve ATCC 15700* demonstrates the production of β-glucosidase, which cleaves glycosides in ginsenoside Rd., generating deglycosylated ginsenoside compound K (Zhong et al., 2016; Zhang et al., 2019). Such metabolic properties position *Bifidobacterium* as a viable option for symbiotic establishment, utilizing its natural product synthesis capabilities for potential therapeutic applications (Zhao et al., 2022).

## 5 The role of biotransformation in uncovering the active substance

The intricate connection between many natural compounds’ poor oral availability and significant pharmacological efficacy is being uncovered by studies on gut microbiota. The challenge lies in the complex structures of glycosides, hindering absorption by intestinal cells and limiting tissue-specific bio-accessibility. These substances undergo microbial enzymatic degradation, transforming into small molecule metabolites that exert diverse impacts on the host (Wardman et al., 2022).

Crucially, the therapeutic effects of natural compounds are significantly influenced by gut microorganisms. This is best illustrated by the conversion of ginsenosides into compound K (CK), which exhibits increased anti-inflammatory, anti-tumor, and lipid-reducing properties (Kim et al., 2013; Kim, 2018). Similarly, curcumin metabolites, influenced by the microbiota, exhibit anti-inflammatory properties through pathways such as PPARγ expression and NF-κB inhibition (Tang et al., 2021; Lu et al., 2022). Urolithin A (UA), a gut microbe-derived compound, showcases neuroprotective and anti-inflammatory properties (Gong et al., 2019; Huang et al., 2022).

Furthermore, the intricate interplay of form, function, composition, etc within the community of gut microbes unveils promising avenues for leveraging natural products. These compounds hold the potential to not only amplify therapeutic efficacy but also mitigate adverse effects, thereby offering a nuanced and refined approach to enhancing health outcomes. Gut microbes can modify the toxicity of certain compounds, such as reducing cardiotoxicity in digoxin metabolism (Kumar et al., 2018). However, harmful substances can also be synthesized by gut microorganisms, emphasizing the need for small molecule inhibitors to regulate specific transformations. Further research is necessary to determine how different doses of natural products affect gut microorganisms and metabolism because excessive consumption can upset the gut microbiota and cause negative effects (Lindell et al., 2022; Zhao et al., 2022).

## 6 Exploring human microbial metabolites

### 6.1 Screening to ecological insights

Diverse organisms, encompassing microorganisms, have long been recognized as prolific producers of secondary metabolites, also known as natural products (Law et al., 2020). Since the middle of the 20th century, the identification of these bioactive compounds, ranging from polyketides (PKs) to nonribosomal peptides (NRPs) and their hybrids, has been predominantly achieved through activity-based testing of cultivatable microorganisms, particularly *Actinomycetes* soil bacteria (Katz and Baltz, 2016). Over time, developments in molecular biology, DNA sequencing, and cultivation techniques have ushered in a new era, enabling the exploration of secondary metabolites from previously unculturable microbes (Lewis et al., 2010; Wilson and Piel, 2013; Sidebottom and Carlson, 2015; Browne et al., 2016). The impact of microbial natural products, as well as their semisynthetic derivatives, on human health, is profound, as they have emerged as vital sources for clinically relevant antibiotics, antifungals, immunosuppressants, anticancer agents, and other pharmaceuticals (Newman and Cragg, 2016). While the primary focus of natural product exploration has historically been medicinal usage, recent research has unveiled the intriguing ecological parts played by secondary metabolites in the organisms that produce them and their broader ecosystems, extending even to the human body (Crawford and Clardy, 2011; Cantley and Clardy, 2015; Wilson et al., 2017).

### 6.2 Secondary metabolites and health implications

**Figure 4:**
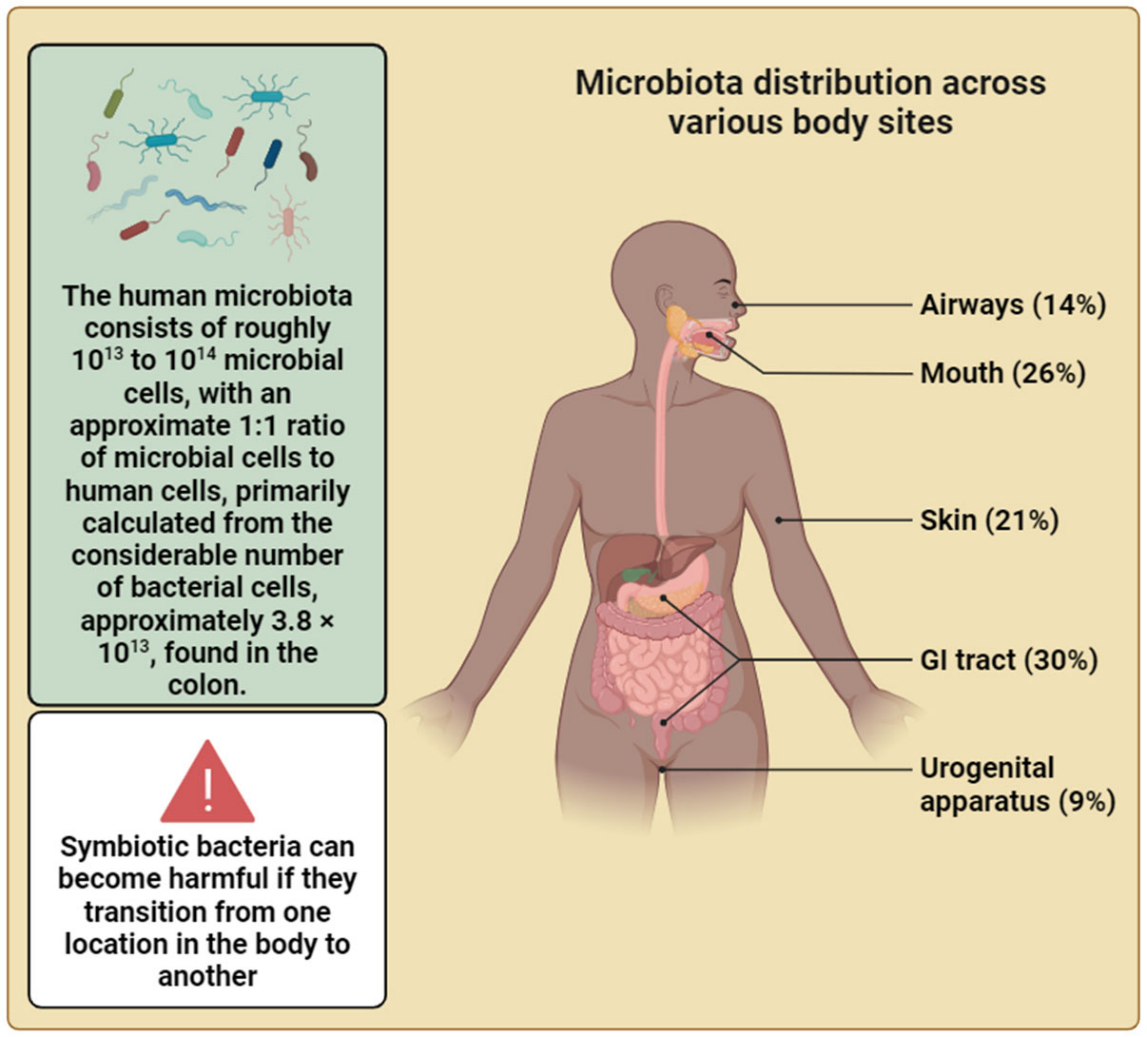
The intricate relationship between humans and their resident microbes is highlighted in this figure. The vast microbial community, comprising bacteria and other microbial organisms, plays a significant part in shaping human health and homeostasis, challenging traditional perceptions of the self as a singular entity. This depiction underscores the profound influence of the microbiota on human physiology and emphasizes the notion that, in the realm of the body’s cellular landscape, we are not alone. Additionally, this depiction underscores the emerging recognition of the gut microbiota as a key orchestrator of systemic health, influencing processes beyond digestion and immunity, including metabolism etc. (Created with Biorender).

The microbiome, consisting of a myriad of microorganisms that inhabit the human body (**Figure 4**), plays a crucial role in shaping human health and influencing various diseases. These microorganisms collectively harbor a gene pool that surpasses the human genome by a factor of 150 (Methé et al., 2012; Weinstock, 2012; Kho and Lal, 2018; Marsh et al., 2023; Walker and Hoyles, 2023). An analysis of data from the Human Microbiome Project (HMP), which includes genomic and metagenomic sequencing, has revealed that bacteria associated with humans possess the genetic machinery necessary to produce an extensive range of secondary metabolites (Donia et al., 2014). Despite this, the identities of the majority of such natural products remain undiscovered, and their functions are not fully comprehended. Investigating the ways through which these molecules impact interactions between hosts and microbes, as well as among different microbial species, could unveil factors that shape the impact of the microbiome on its host. This exploration holds the potential to yield targeted therapeutic options and may serve as a wellspring of innovative approaches to address human diseases. Few methods for uncovering natural products, and demonstrating their individual or collaborative use in deciphering the intricate metabolic interactions within the human microbiome have been presented (Wilson et al., 2017; Milshteyn et al., 2018).

#### 6.2.1 Exploring biological functionality in metagenomic libraries through functional metagenomics

Microbial organisms exist widely in nature, thriving in diverse environmental conditions. Many of them cannot be cultured conventionally. Metagenomics allows the exploration of microbial communities directly from environmental samples, regardless of culturability. This method unveils species diversity and offers insights into their functions in natural settings. By cloning and expressing metagenomic DNA in a different host, function-based screenings can uncover novel proteins with industrial applications from previously inaccessible microorganisms. Functional metagenomics holds promise in industries like food and pharmaceuticals, facilitating the discovery of enzymes suitable for various processing conditions, novel bioactives including antimicrobial agents, and addressing concerns like development of antibiotic resistance (Coughlan et al., 2015).

Natural products have long played a pivotal role in the development of therapeutics for a variety of diseases. Traditionally, soil and marine environments have provided a rich reservoir from which diverse chemical scaffolds could be discovered. Recently, the human microbiome has been recognized as a promising niche from which secondary metabolites with therapeutic potential have begun to be isolated. The expansive history of identifying bacterial natural products in other environments is informing the approaches being brought to bear on the study of the human microbiota have also been addressed in many studies. These tools can lead to insights about microbe-microbe and host-microbe interactions and help generate biological hypotheses that may lead to developments of new therapeutic modalities (Milshteyn et al., 2018).

The functional metagenomic strategy seeks to explore bacterial metabolites through the systematic screening of metagenomic libraries for distinct bioactivities. Upon identifying a bioactive clone through functional screening, isolation of the clone and subsequent sequencing can pinpoint the gene or set of genes involved in producing the active metabolite. This direct association between the metabolite and its biosynthetic components is a key advantage of functional metagenomics. In the realm of the human microbiome, where a substantial portion of bacterial species have undergone full sequencing, this approach facilitates the simultaneous linking of bioactive metabolites with their functions inside the host and their producers within the Microbiome (Milshteyn et al., 2018).

Functional metagenomics can be applied in many different ways, but the most successful approaches that involve human microbiome metagenomic libraries have employed testing culture broth filtrates from metagenomic clones that are individually arrayed against assays using human cell reporter assays. In an early endeavor, Lakhdari and colleagues utilized a nuclear factor-kB (NF-kB) activation screen to identify 171 clones from a 2,640-clone human gut microbiome fosmid library, showing modulation of NF-kB reporter activity (Lakhdari et al., 2010). NF-kB, a rapidly inducible transcription factor with wide involvement in diverse cellular responses, is notably implicated in the immuno-inflammatory response in the gut, rendering it a suitable indicator for detecting various microbe-host interactions (Zhang et al., 2017).

In groundbreaking research, Cohen et al. (2015) (Cohen et al., 2015) employed functional metagenomics to identify host-associated bacterial effector genes, leading to the discovery of commendamide, a novel N-acyl amide. Commendamide was found to activate the GPR132/G2A receptor, implicated in immune cell functions. Subsequent research (Cohen et al., 2017) expanded the repertoire of N-acyl amides from the human microbiome, revealing structural similarities to endogenous GPCR ligands. Notably, certain bacterial GPR119 agonists mirrored the effects of endogenous ligands, regulating metabolic hormones and glucose homeostasis in mouse models. This suggests a potential for microbiome-biosynthetic gene therapy as a novel therapeutic modality. These studies highlight how functional metagenomics unveils microbiome-host interactions, identifies therapeutic targets, and explores microbiota-derived small molecules for innovative therapeutic strategies (Milshteyn et al., 2018).

#### 6.2.2 Methods for extracting natural products through sequence-guided exploration of (Meta) genomes

Genome mining represents a computational approach to small-molecule discovery, relying on algorithmic predictions to determine biosynthetic gene clusters (BGCs) within sequence data. Established algorithms, such as antiSMASH, NP.searcher, MultiGeneBlast, etc predominantly utilize conserved sequences from extensively studied BGC groups to pinpoint novel gene clusters within these families (Medema et al., 2013; Reddy et al., 2014; Medema and Fischbach, 2015). Recent developments, exemplified by ClusterFinder, aim to extend this capability to discover previously unknown BGC groups (Cimermancic et al., 2014). The detailed examination of the computational complexities associated with sequence-guided natural product identification has been extensively explored in other sources (Medema and Fischbach, 2015), our focus here centers on the application of sequence-based methods for bacterial metabolite identification within the human microbiome (Milshteyn et al., 2018).

Over the last decade, significant initiatives have been undertaken to sequence and annotate human microbiome bacteria, driven by a keen interest in understanding the microbiota’s impact on human health. This has resulted in a remarkable wealth of sequence data unique to the human microbiome, encompassing over 3,000 full and partial reference genomes, along with numerous (meta)genomic shotgun sequencing datasets from both healthy and sick individuals (Aagaard et al., 2013; Donia et al., 2014). Utilizing this data, researchers are currently employing bioinformatics tools to uncover new secondary metabolite BGCs within the human microbiome. The overarching objective of these bioinformatics-driven discovery endeavors is to elucidate and functionally characterize the metabolites that are encoded by recently identified natural product BGCs (Milshteyn et al., 2018).

Utilizing the ClusterFinder algorithm, researchers identified over fourteen thousand biosynthetic gene clusters (BGCs) in the Human Microbiome Project (HMP) data, revealing the prevalence of various small molecule families, including thiopeptides (Cimermancic et al., 2014). Lactocillin, a novel thiopeptide antibiotic produced by the vaginal isolate *Lactobacillus gasseri JV-V03*, demonstrated potent activity against pathogenic bacteria (Mullane et al., 2015). Another innovative approach, involving the synthesis of bioinformatically predicted natural product-like structures termed syn-BNPs (Chu et al., 2016), led to the discovery of humimycins with broad-spectrum antibiotic activity. Genome mining also uncovered a family of 47 nonribosomal peptide synthetase (NRPS) BGCs present in over 90% of HMP stool samples, producing pyrazinones and dihydropyrazinones (Guo et al., 2017). Additionally, the exploration of mucin-utilizing bacteria and the identification of the fldAIBC cluster shed light on a specific bacterial metabolic pathway involving indoleacrylic acid, linking it to protective effects in a colitis model and proposing a connection between a healthy mucus layer, anti-inflammatory metabolites, and inflammatory bowel disease (IBD) (Wlodarska et al., 2017). These findings highlight the potential of mining the human microbiome for novel therapeutics and insights into host-microbe interactions (Milshteyn et al., 2018).

#### 6.2.3 Exploring chemistry-centric strategies for uncovering natural products in the human microbiome

Many instances arise where genome-centric approaches are not the primary focus, highlighting the diverse avenues of research that extend beyond the realm of genomics. Essentially, both pathogenic and nonpathogenic bacteria have had their secondary metabolites studied for many years, predominantly through laboratory cultivation and analytical chemistry. The examination of individual bacteria *in vitro* has yielded a considerable array of compounds, with varied degrees of intricacy and biological significance, and these have been thoroughly reviewed in the latest research (Donia and Fischbach, 2015; Mousa et al., 2017). With the current intense scientific exploration of the human microbiome, there is a growing interest in delving into deeper biological contexts for *in vitro*-discovered secondary metabolites. This aspect has not always been a forefront consideration in traditional natural product discoveries. Increasingly, robust foundations of chemistry-centric discovery techniques are now employed to formulate intriguing biological hypotheses based on identified natural products. Strong underpinnings of chemistry-focused discovery methods are increasingly being employed to formulate intriguing biological theories rooted in discovered natural compounds (Milshteyn et al., 2018).

Utilizing sophisticated analytical methods, like liquid chromatography-mass spectrometry (LC-MS), has proven instrumental in dissecting bacterial metabolites and unraveling their functional roles. For instance, research in 2009 demonstrated that Lactobacillus plantarum, a beneficial probiotic, might reduce inflammation both *in vivo* and *in vitro* by regulating NF-kB (Petrof et al., 2009; Van Baarlen et al., 2009; Bansal et al., 2010). Zvanych et al.’s (2014) (Zvanych et al., 2014) study further applied principal component analysis to LC-MS data, identifying dipeptide molecules with pyroglutamic rings, like pyro-phenylalanine and pyro-tryptophan, which, when introduced into mice, resulted into reduced splenic synthesis of IFN-g. Another notable approach involves the functional evaluation of secreted bacterial metabolites, as illustrated by the discovery of lugdunin, a novel antibiotic from nasal bacteria. Metabolomics platforms, especially those employing HRMS (High-Resolution Mass Spectrometry), have performed an essential part in examining the chemical fingerprint of the human microbiota, offering insights into diverse biological activities and potential biomarkers, as seen in studies comparing germ-free and colonized mice, cardiovascular health, and neurological diseases in mouse models (**Table 2**) (Wikoff et al., 2009; Hsiao et al., 2013; Brown and Hazen, 2017; Hou et al., 2022). While challenges persist in identifying unknown secondary metabolites, the evolving strategies for HRMS data collection and analysis provide optimism for unearthing therapeutic leads in the future (Peisl et al., 2018).

**Table 2:** Microbiota research: insights from murine models (Hou et al., 2022).

	Murine models	Area of Study	Importance
**1.**	Sterile mice populated with human microbial communities	Explore the nuanced relationship between host and microbiota in various systems, such as the gastrointestinal tract, cardiology, reproductive biology, lipid metabolism, and bone homeostasis. Investigate the dynamic interplay within each of these physiological contexts.	Create an environment devoid of any microorganisms and facilitate the introduction of particular microbiota, while simultaneously modifying the typical physiological parameters of the host.
**2.**	Chemically altered mice	Utilizing chemical agents to harm the epithelial cells of the gut or trigger an immune response within the mucosal lining.	A frequently employed method to instigate colitis in mice. May yield inconsistent outcomes due to differences in experimental design and environmental variables.
**3.**	Drug-induced mice	Antibiotics have the capacity to selectively reduce certain members of the microbiota, enabling the investigation of the bacteria's involvement in sustaining cellular functionality and signaling pathways post-development.	Relevant for any mouse genotype or condition. Could lead to the emergence of drug-resistant bacteria.
**4.**	Genetically engineered mice	Mimic the observable characteristics linked to genetic abnormalities in conditions like Inflammatory Bowel Disease (IBD).	Offers a potent tool for investigating the pathological processes of human illnesses. The presence of genes participating in multiple pathways could potentially impact the outcomes.

## 7 Conclusion

The exploration of the human microbiome and its intricate relationship with natural products has unveiled a captivating realm within the field of biology. The human digestive system, teeming with a diverse microbiota, serves as a dynamic ecosystem comprising an astounding number of microorganisms. This microbial assembly, primarily constituted of anaerobic bacteria, orchestrates processes finely tuned to human physiology, functioning as an organ with a genetic repertoire surpassing the human genome. Among its pivotal roles is the breakdown of indigestible dietary components, highlighting its indispensable contribution to our overall well-being.

**Figure 5:**
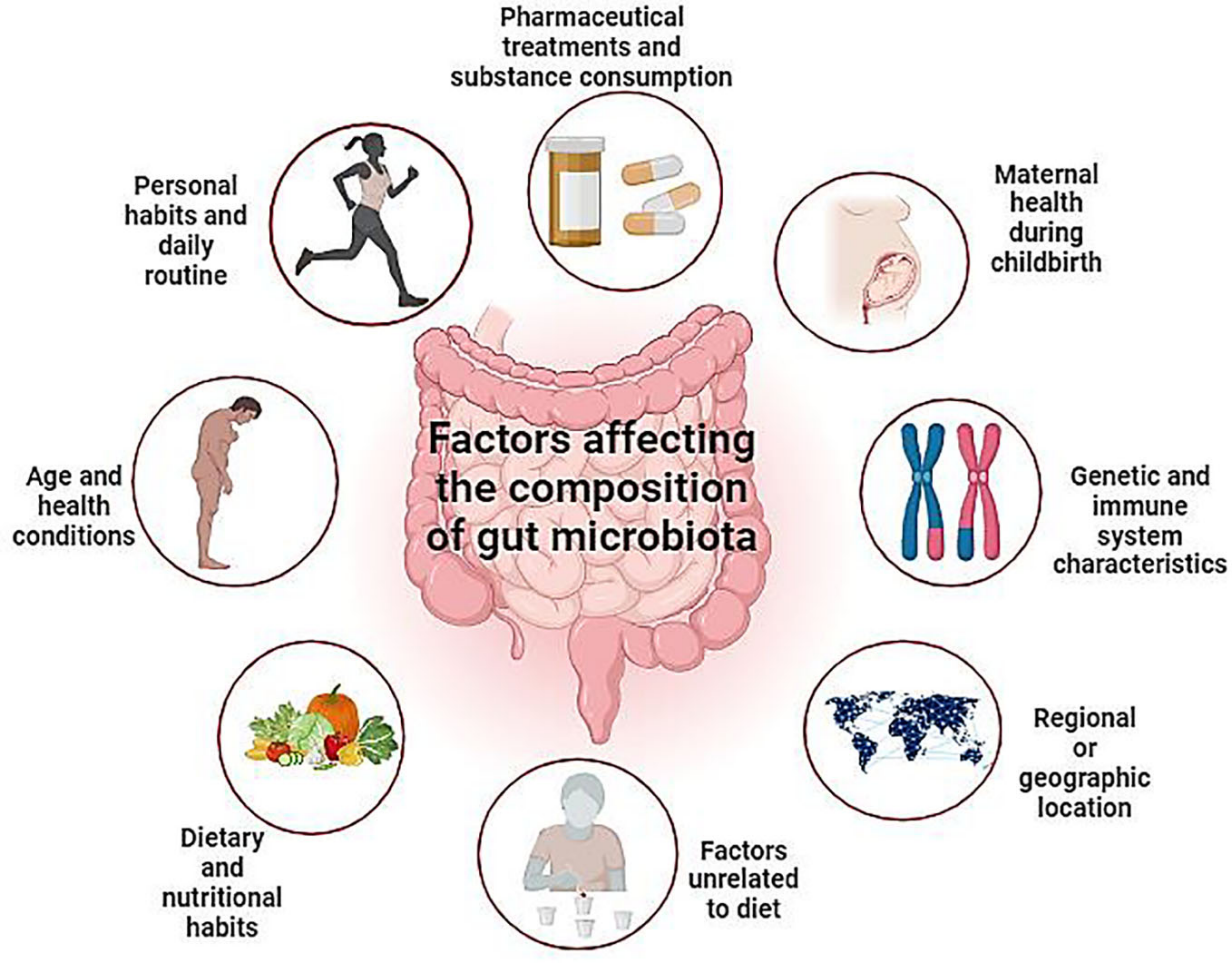
Depicts diverse elements influencing gut microbiota modulation, encompassing lifestyle, age, medication, genetics, geography, and dietary factors. This comprehensive overview highlights the multifaceted nature of gut microbiota dynamics. By highlighting these intricate relationships, this visual representation enhances our understanding of the dynamic interplay between external influences and internal microbial ecosystems, ultimately informing personalized approaches to gut health management.” (Created with Biorender).

The impact of the microbiome extends beyond mere digestion, permeating into complex physiological processes, influencing immune system modulation, and perhaps aiding in the growth of the CNS and brain (**Table 3**) (Ghosh et al., 2021). Mouse models have underscored the vital role of a healthy bacterial environment in maintaining normal physiological processes, while dysbiosis has been associated with an array of diseases. Beyond dietary influences, gut microbiota is significantly affected by various factors (**Figure 5**) and exhibits rapid responses to system alterations due to the interplay between genetic and environmental factors. The burgeoning field of microbiome research is evidenced due to the increase in clinical studies and investments, emphasizing its potential to revolutionize healthcare. There is increasing evidence linking host-associated bacteria to both healthy development and disease, but there is still a significant knowledge vacuum regarding the precise mechanisms by which bacterial activities impact the physiology of mammals or microbiomes. The traditional molecular biology dogma falls short of elucidating the ultimate outcomes of information transfer within a biological system, particularly when it comes to small molecules and natural products.

**Table 3:** Compilation of microbial metabolites, gut microbiota, and their roles (Ghosh et al., 2021).

	Microbial-derived metabolites	Assocaited Gut Flora/microbiota	Role(s)
1.	Amino acids (like lysine etc)	*S. aureus, E. coli, P. aeruginosa* etc.	➢ SOS response: Triggering ➢ Biofilm formation: Modulation ➢ Deamination ➢ Regulating peptidoglycan synthesis
2.	Derivatives of indole (like indole-3-propionic acid, melatonin etc)	*C. sporogenes, E. coli*	➢ Regulation of the intestinal barrier. ➢ Control of endothelial dysfunction etc.
3.	Vitamins (like vitamin Kz2, B2 etc)	*Bifidobacterium, S. aureus*, etc.	➢ Involvement in redox cycling. ➢ Protection against pathogens. ➢ Facilitation of DNA replication, methylation, and repair. ➢ Synthesis of vitamins, nucleotides, and amino acids.
4.	Short-chain fatty acids (butyrate, valerate, acetate etc)	*Bifidobacterium* sp.*, Coprococcus, Clostridium, Bacteroidetes* etc	➢ Regulation of host metabolic pathways via cell signaling. ➢ Modulation of the immune system. ➢ Sustaining energy balance. ➢ Enhanced glucose tolerance and insulin sensitivity. ➢ Regulation of osmotic balance.
5.	Bile acid metabolites (like cholic acid, deoxycholic acid, taurocholic acid etc)	*Clostridium, Lactobacillus* etc	➢ Activation of nuclear receptors and cellular signaling pathways in the host. ➢ Demonstrating antimicrobial properties. ➢ Regulation of lipid absorption.

Gut microorganisms perform a pivotal function in decomposing and transforming natural products, generating a diverse array of metabolites and functional compounds. The chemical transformations involved significantly regulate the form of natural product substrates. Importantly, the impact of gut microbiota extends beyond structural modifications, influencing the chemical landscape, pharmacological activities, and metabolic mechanisms of natural products. Intriguingly, the perspective of harnessing gut microorganisms for extensive manufacturing of active metabolites and compound synthesis remains largely unexplored. The study of gut microorganisms, their metabolites, and the intricate reactions concerned with the dynamic interplay between natural products and gut microbiota holds immense importance. Unraveling these interactions is not only crucial for understanding pharmacological mechanisms but also for unlocking the untapped potential of natural products in various applications.

## 8 Future perspectives

Looking ahead, the field stands at the precipice of unprecedented discoveries and applications. Delving into the study of gut microbes and their role in the synthesis of bioactive compounds on a larger scale presents an exciting avenue for future research. The untapped potential of harnessing the therapeutic benefits of these interactions holds promise for innovative approaches to medicine and therapeutics. The multifaceted relationships between natural products and gut microbiota open new doors for drug discovery, as the microbiome becomes a potential source of novel compounds with therapeutic applications. Future research should focus on elucidating the specific mechanisms by which these microbiome-derived naturally occurring compounds exert their effects on the well-being of humans. This involves a deeper understanding of the metabolic pathways involved, the pharmacological activities of these compounds, and their potential applications in treating various diseases.

Moreover, the development of technologies and methodologies for the systematic characterization of small molecules generated by human-associated microorganisms is crucial. Advancements in analytical techniques and high-throughput screening methods will facilitate the identification and isolation of novel microbiome-derived natural products. Collaborations between microbiologists, pharmacologists, and clinicians will be essential in translating these discoveries into practical applications. The integration of microbiome-based therapies into personalized medicine holds the potential to revolutionize healthcare, offering tailored treatments based on an individual’s unique microbiome profile. In a nutshell, the intersection of natural products, bacteria, and human health represents a frontier of scientific exploration with profound implications for medicine and therapeutics. As we navigate this dynamic field, the collaborative efforts of researchers across disciplines will be instrumental in unlocking the full potential of microbiome-derived natural products and ushering in a new era of innovative healthcare solutions.

## Author contributions

HQ: Conceptualization, Writing – original draft, Writing – review & editing. AHS: Writing – review & editing. AA: Funding acquisition, Writing – review & editing. MM: Conceptualization, Funding acquisition, Project administration, Supervision, Writing – review & editing. 
